# Cognitive therapy and interpersonal psychotherapy reduce suicidal ideation independent from their effect on depression

**DOI:** 10.1002/da.23151

**Published:** 2021-03-23

**Authors:** Jaël S. van Bentum, Suzanne C. van Bronswijk, Marit Sijbrandij, Lotte H. J. M. Lemmens, Frenk F. P. M. L. Peeters, Marjan Drukker, Marcus J. H. Huibers

**Affiliations:** ^1^ Department of Clinical, Neuro‐ and Developmental Psychology, Amsterdam Public Health Research Institute Vrije Universiteit Amsterdam Amsterdam The Netherlands; ^2^ Department of Clinical Psychological Science, Faculty of Psychology and Neuroscience Maastricht University Maastricht The Netherlands; ^3^ World Health Organization Collaborating Centre for Research and Dissemination of Psychological Interventions Vrije Universiteit Amsterdam Amsterdam The Netherlands; ^4^ Department of Psychiatry and Psychology, School for Mental Health and Neuroscience, Faculty of Health, Medicine and Life Sciences Maastricht University Maastricht The Netherlands; ^5^ Department of Psychology University of Pennsylvania Philadelphia Pennsylvania USA

**Keywords:** adult, cognitive behavioral therapy, depression, interpersonal psychotherapy, linear models, randomized controlled trial, suicide

## Abstract

**Background:**

Clinical guidelines suggest that psychological interventions specifically aimed at reducing suicidality may be beneficial. We examined the impact of two depression treatments, cognitive therapy (CT) and interpersonal psychotherapy (IPT) on suicidal ideation (SI) and explored the temporal associations between depression and SI over the course of therapy.

**Methods:**

Ninety‐one adult (18–65) depressed outpatients from a large randomized controlled trial who were treated with CT (*n* = 37) and IPT (*n* = 54) and scored at least ≥1 on the Beck Depression Inventory II (BDI‐II) suicide item were included. Linear (two‐level) mixed effects models were used to evaluate the impact of depression treatments on SI. Mixed‐effects time‐lagged models were applied to examine temporal relations between the change in depressive symptoms and the change in SI.

**Results:**

SI decreased significantly during treatment and there were no differential effects between the two intervention groups (*B* = −0.007, *p* = .35). Depressive symptoms at the previous session did not predict higher levels of SI at the current session (*B* = 0.016, *p* = .16). However, SI measured at the previous session significantly predicted depressive symptoms at the current session (*B* = 2.06, *p* < .001).

**Conclusions:**

Both depression treatments seemed to have a direct association with SI. The temporal association between SI and depression was unidirectional with SI predicting future depressive symptoms during treatment. Our findings suggest that it may be most beneficial to treat SI first.

## 1 INTRODUCTION

Suicide is a global health problem causing over 800,000 people to die each year (World Health Organization, 2014). In 2019, 1.811 individuals died by suicide in the Netherlands alone (Centraal Bureau voor de Statistiek, 2020). Suicide can be perceived as a continuum that ranges from suicidal ideation (SI; feeling life is not worth living or thoughts of ending one's life), to suicide attempts (self‐injurious behavior with the intent to cause death), to death by suicide (self‐inflicted and intentional killing of oneself; Devenish et al., 2016).

Research over the past years concluded that treatment may be most effective when directly targeting suicidal behavior or thoughts (Mewton & Andrews, 2016; Tarrier et al., 2008). However, the clinical guidelines for treating suicidality often suggest psychotherapies with the primary psychiatric diagnosis (e.g., major depressive disorder [MDD]) as the main treatment focus and report few recommendations regarding the selection of treatment (Bernert et al., 2014; Jacobs et al., 2010). A lack of evidence remains about which psychotherapeutic approach is most beneficial to directly target suicidality. Randomized controlled trials (RCTs) of psychotherapies for mental illnesses or suicidality showed high between‐study heterogeneity due to differences in suicidal phenotypes (ideation, attempts, nonsuicidal self‐injury, etc.), type of treatments, and diagnoses (Calati & Courtet, 2016).

Most clinicians are unaware that suicide risk should be addressed specifically but assume that if the primary psychiatric disorder is treated, the suicidal symptoms will follow (Sudak & Rajalakshmi, 2018). There seems to be an explicit link between depression and suicide risk as recurring thoughts of death are one of the nine symptoms characterizing MDD (American Psychiatric Association, 2013). Nonetheless, while the risk of a suicide attempt is almost eight‐fold during an MDD episode compared with a period of full remission (Sokero, 2003), it is not yet clear whether depression treatments *directly* decrease SI. A recent meta‐analysis examining the effects of psychotherapy for depressed adults found that only 3 out of 1344 studies reported outcomes related to suicidal cognitions or behaviors. In these studies, the effect of treatments on SI and suicide risk was small and nonsignificant (Cuijpers et al., 2013).

Currently, the two most commonly practiced psychological treatments for depression are cognitive behavior therapy (CBT) and interpersonal psychotherapy (IPT). Studies show inconclusive results about whether CBT for depression is effective in the treatment of suicidal symptoms. In a recent study, male veterans experienced a 52.6% decrease in SI severity throughout CBT for depression (Kumpula et al., 2019). Another study among depressed adolescents showed that CBT interventions were at least as efficacious as pharmacotherapy in reducing suicidality (Devenish et al., 2016). For now, there is insufficient trial evidence to suggest that CBT for depression also leads to reduced suicidal cognitions and behaviors (Mewton & Andrews, 2015; Watts et al., 2012).

Even less evidence is available on the efficacy of IPT in reducing SI. In one study, school‐based IPT for adolescent depression showed a reduction in SI (and depression) when compared with treatment as usual (Tang et al., 2009), and IPT appeared to be a safe treatment with past suicide attempters (Rucci et al., 2011). While these findings suggest that IPT might be a valuable treatment for SI in depressed patients, more rigorously designed RCTs are needed to increase the evidence base.

A recent study by Weitz et al. (2014) examined the clinical effects of CBT, IPT, pharmacotherapy, and placebo on suicidality using the suicide item on the Beck Depression Inventory‐II (BDI‐II; Beck et al., 1996) and Hamilton Rating Scale for Depression (HRSD; Hamilton, 1960). No differences among active treatment groups and placebo were found on the BDI‐II suicide item. Only IPT and antidepressant medications showed a reduction in SI (relative to placebo) on the HRSD. Furthermore, the effect of treatment on change in SI disappeared once change in depression was controlled for, suggesting that decreased suicidality goes together with decreased depression scores.

While these findings suggest that depression and suicidality are related, theories about the true nature of the relationship between the two are still in their infancy (Oquendo & Currier, 2009), as is a possible temporal relation between the two during therapy. Research has mostly focused on the effectiveness of treatments, but barely on whether depressive symptoms decrease before suicidality or the other way around. As mentioned, clinicians often assume that if the depression is treated successfully, suicidal symptoms will automatically disappear as well, leaving suicidality a secondary focus of treatment. Therefore, in addition to examining the effectiveness of psychotherapies for depression on suicidality, understanding the temporal relationship between the two constructs could have great clinical implications.

This study examined the impact of two depression treatments (cognitive therapy [CT] and IPT) on SI in the acute phase of MDD. We built upon the current research by using session‐by‐session ratings of both depression severity and SI, allowing us to explore the dynamic and individual course of SI (within the context of treatment of depression) over time more accurately using multilevel modeling. The current study had the following aims. First, we tested whether CT and IPT outperformed the wait‐list control (WLC) condition by comparing change in BDI‐II suicide item scores of patients after 8 weeks. We hypothesized that CT and IPT would show a greater reduction in BDI‐II suicide item scores compared with WLC. Second, we tested whether IPT and CT would lead to decreased SI and whether the effects would still hold after controlling for depression severity at each session in the course of therapy. We also tested whether one of the treatments was superior to the other in this potential reduction in SI. Based on results of the main trial and limited research available, we hypothesized that CT and IPT would not differ in reducing SI. Third, we explored the associations over time between depressive symptoms and SI. Using mixed effects time‐lagged models we examined whether depressive symptoms decreased before SI or the other way around. In other words, we examined whether depressive symptoms at the previous session (1‐week lag) was associated with current SI, and whether SI at the previous session (1‐week lag) was associated with current depressive symptoms. Given that, to our knowledge, this was the first study to evaluate the temporal relation between SI and depression, we did not have a hypothesis about the direction of this relation.

## 2 METHODS

### 2.1 Study design

Data were collected as part of a large single‐center RCT examining the clinical effects of individual CT and IPT for MDD, compared with a WLC condition. Assessments of both depression severity and SI were administered at the start of each therapy session. Details are described elsewhere (Lemmens et al., 2019), and therefore will only be briefly summarized here.

### 2.2 Participants and recruitment

**Figure 1:**
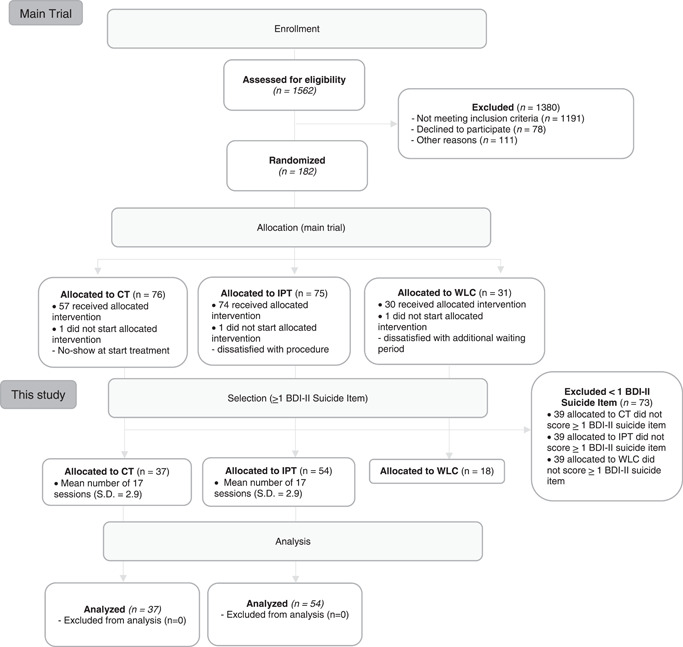
Patient flow chart. BDI‐II, Beck Depression Inventory II; CT, cognitive therapy; IPT, interpersonal psychotherapy; WLC, wait‐list control

Individuals with a primary diagnosis of MDD (as confirmed by trained clinicians using the Structured Clinical Interview for DSM‐IV Axis I disorders (SCID‐I; First et al., 1995) were recruited from the mood disorders unit of the Maastricht Outpatient Mental Health Center (RIAGG Maastricht). Additional inclusion criteria were: internet access, an e‐mail address, and Dutch language proficiency. Exclusion criteria were: bipolar or highly chronic depression (current episode > 5 years), acute suicide risk warranting crisis intervention or services more intensive than weekly psychotherapy, concomitant pharmacological or psychological treatment, substance abuse, and mental retardation (IQ < 80). A total of 182 outpatients, aged 18–65 years, were included and randomly allocated to one of three conditions: CT (*n* = 76), IPT (*n* = 75), or an 8‐week WLC condition followed by treatment of choice (CT or IPT, *n* = 31). Randomization occurred through an automated computer program and was pre‐stratified according to presence of previous episodes. In this study, we limited the sample to individuals who score at least ≥1 on the BDI‐II suicide item at baseline (*n* = 109) (cf. Weitz et al., 2014). When analyzing the second research question only the active therapy groups (CT and IPT; *n* = 91, CT: *n* = 37; IPT: *n* = 54) were used (see Figure 1).

### 2.3 Ethical approval

The study was approved by the Medical Ethics Committee of Maastricht University Medical Center, and registered at the Netherlands Trial Register (ISRCTN 67561918).

### 2.4 Interventions

CT and IPT were described in protocols following guidelines by Beck et al. (1979) for CT and guidelines by Klerman et al. (1984) for IPT. Each intervention consisted of 16–20 sessions of 45 min (17 sessions average, *SD* = 2.9 for both interventions; Lemmens et al., 2015). Sessions were scheduled weekly and allowed to be planned less frequently toward the end of therapy. Treatments were performed by 10 licensed psychologists, psychotherapists, and psychiatrists (five in each condition) with an average 9.1 years of clinical experience (*SD* = 5.4; range, 4–21 years). See Lemmens et al. (2015) for details regarding treatment integrity.

### 2.5 Measures

The primary outcome, severity of suicidality (ideation through intent), was measured with the suicide item on the BDI‐II (item 9; Beck et al., 1996). The BDI‐II is a 21‐item self‐report questionnaire measuring depressive symptom severity over the past 2 weeks and has strong psychometric properties (Wang & Gorenstein, 2013). Each item is rated on a 4‐point scale (0–3) with higher scores indicate higher levels of severity (0–63). The suicide item measures moderate SI through intent (0—*I do not have any thoughts of killing myself*, 1—*I have thoughts of killing myself, but I would not carry them out*, 2—*I would like to kill myself*, 3—*I would kill myself if I had the chance*). A recent study has established the predictive validity of the item as a SI screening tool (Green et al., 2015). The BDI‐II was assessed at baseline and the start of each therapy session throughout treatment. For depressive symptoms, the BDI‐II total score of each session excluding the suicide item was used, and for SI the suicide item of each session was used.

### 2.6 Statistical analyses

Patient characteristics were explored using descriptive statistics stratified by SI and condition (*n* = 182). Using an independent samples *t* test (two‐sided), we examined whether the active treatment groups (CT and IPT combined) outperformed WLC by comparing change in BDI‐II suicide item scores of patients after 8 weeks. We then limited the sample to only the active treatment groups since we were interested in the effects of treatment for depression on SI ratings during treatment (*n* = 91).

All analyses were performed using intention‐to‐treat. Primary outcome was the BDI‐II suicide item score at baseline and the start of each therapy session. Data had a two‐level structure with multiple assessments per person and were ideally suited for mixed‐effects analyses (see Supporting Information I). The following fixed effects were included in the model: time (number of sessions), treatment (centered at CT = −0.5 or IPT = 0.5) and the other depressive symptoms (total BDI‐II score excluding suicide item at each session, as a time varying covariate). The difference between CT and IPT was represented by the “time × treatment” interaction.

Next, we performed two mixed‐effects time‐lagged models to examine a temporal relation between the change in other depressive symptoms and the change in SI. First, we examined whether other depressive symptoms at the previous session (depression—1 week) predicted current SI. Included fixed effects were time (number of sessions), and depression score at the previous session (as the lagged predictor variable). Second, we examined whether SI measured at the previous session (SI—1 week) predicted current depressive symptoms. For this model, included fixed effects were time (number of sessions), and suicide item score of previous session (as the lagged predictor variable). Mixed analyses were performed using STATA (version 16·0). Other analyses were carried out in SPSS (version 25).

## 3 RESULTS

### 3.1 Description of the sample

Characteristics of the sample stratified by SI and condition (CT, IPT, or WLC) are displayed in Table 1. Forty percent of the sample did not experience SI according to the BDI‐II at the start of the RCT and were excluded from this study. Overall baseline scores on the suicide item were low, where 99 participants scored 1—*I have thoughts of killing myself, but I would not carry them out*, 7 scored 2—*I would like to kill myself* and only 3 scored 3—*I would kill myself if I had the chance*. There were no relevant differences between patients in the active treatment groups and the WLC condition for sociodemographic variables.

**Table 1:** Characteristics of the sample comparing heightened suicidal ideation (*n* = 109) patients with nonsuicidal ideation patients (*n* = 73) stratified according to condition

	Cognitive therapy		Interpersonal psychotherapy		Waiting list control	
	Heightened suicidal ideation	No suicidal ideation	Heightened suicidal ideation	No suicidal ideation	Heightened suicidal ideation	No suicidal ideation
*N*	37	39	54	21	18	13
*Sociodemographic variables*						
Female sex, *n* (%)	23 (62.2)	31 (79.5)	32 (59.3)	14 (66.7)	8 (44.4)	8 (61.5)
Age (years), mean (*SD*)	40.78 (11.92)	41.56 (12.94)	41.02 (11.74)	42.05 (12.27)	39.78 (11.73)	33.23 (12.95)
Education, *n* (%)						
Low	8 (21.6)	8 (20.5)	10 (18.5)	3 (14.3)	7 (38.9)	1 (7.7)
Medium	21 (56.8)	27 (67.2)	28 (51.9)	13 (61.9)	9 (50.0)	11 (84.6)
High	8 (21.6)	4 (10.3)	16 (29.6)	5 (23.8)	2 (11.1)	1 (7.7)
Partner, yes, *n* (%)	15 (40.5)	28 (71.8)	35 (64.8)	16 (76.2)	11 (61.1)	7 (53.8)
Active employment, yes, *n* (%)	23 (62.2)	20 (51.3)	38 (70.4)	9 (42.9)	13 (72.2)	11 (84.6)
Clinical features BDI‐II						
BDI‐II suicide item score baseline, mean (*SD*)	1.14 (0.48)	0	1.13 (.39)	0	1.06 (0.236)	0
BDI‐II score baseline, mean (*SD*)	30.73 (10.3)	26.18 (6.9)	32.65 (7.9)	27.33 (10.39)	32 (10.7)	25.31 (9.5)

*Note*: Heightened suicidality = score of ≥1 on suicide item of Beck Depression Inventory II.

Abbreviation: BDI‐II, Beck Depression inventory II.

### 3.2 CT and IPT versus waiting list

**Figure 2:**
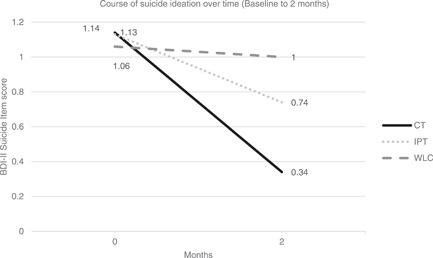
Suicidal ideation over time (baseline to 2 months) in waiting list condition (WLC) versus active condition (cognitive therapy [CT] and interpersonal psychotherapy [IPT])

Patients in the WLC condition showed minimal changes on the suicide item score across the 8‐week waiting list period suggesting that there was no spontaneous recovery (Figure 2). An independent samples *t* test showed a significantly larger improvement on the mean BDI‐II suicide item in the active treatment conditions *(M *=* *−0.54, *SD* = 0.65*)* after 8 weeks of therapy compared with 8 weeks of receiving no treatment in the WLC condition, *M* = −0.06, *SD* = 0.43, *t*(100) = −2.94, *p* = .004, 95% confidence interval, −0.81 to −0.16.

### 3.3 Effects of CT and IPT on SI

**Figure 3:**
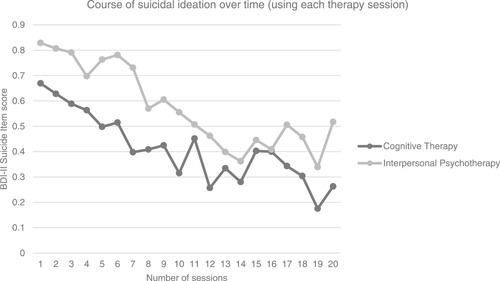
Suicidal ideation over time using each therapy session (cognitive therapy vs. interpersonal psychotherapy–model‐based estimated means of each session). BDI‐II, Beck Depression Inventory II

Table 2 shows the final mixed‐effects model on the primary outcome. The time × condition interaction was nonsignificant (*B* = −0.007, *p* = .35), indicating no differential effects in BDI‐II suicide item reductions between the two treatment groups. The analysis of the basic model (time, condition, time × condition) on the BDI‐II suicide score showed a significant main effect of time, indicating that SI decreased significantly during treatment, irrespective of the severity of suicidality or other depressive symptoms at baseline. A graphic representation of change in SI over time as measured with the suicide item using model‐based estimated means is shown in Figure 3.

**Table 2:** Results of mixed‐effect model estimating the effects of CT and IPT on suicidal ideation as repeatedly measured by the suicide item Beck Depression Inventory‐II during treatment

	Fixed effects			Random effects	
	Coef.	*SE*	*p*	Var.	*SE*
Intercept	−0.031	0.052	.55	0.11	0.031
BDI‐II excluding suicide item (time‐varying variable)	0.12	0.010	<.001		
Condition	0.074	0.008	.35		
Time	−0.026	0.0041	<.001	0.0007	0.00021
Time × Condition	−0.031	0.052	.55		

Abbreviations: BDI‐II, Beck Depression Inventory second edition; BDI‐II Baseline severity, standardized BDI‐II score minus suicide item at baseline; CT, cognitive therapy; IPT, interpersonal psychotherapy; Time, time was centered at the end of treatment (session 20); Var., variance.

### 3.4 Temporal relation between depressive symptoms and SI

Table 3 provides the estimates of the mixed‐effect time‐lagged models examining the temporal relation between other depressive symptoms and SI over the course of treatment. Results of the first model (“depression previous session,” “time”) indicated that depressive symptoms at the previous session did not predict higher levels of SI at the current session (*B* = 0.016, *p* = .16). However, results of the second model showed that, when controlling for depression severity at baseline, SI measured at the previous session significantly predicted depressive symptoms at the current session (*B* = 2.06, *p* < .001).

**Table 3:** Results of mixed‐effect time‐lag models examining the temporal connection between depressive symptoms and suicidality as repeatedly measured by the Beck Depression Inventory‐II during treatment

	Fixed effects			Random effect			Fixed effects			Random effects	
Outcome: Current suicidality	Coef.	*SE*	*p*	Var.	*SE*	Outcome: Current depression	Coef.	*SE*	*p*	Var.	*SE*
*Depression previous session*	0.0016	0.0011	.156			*Suicidality previous session*	2.063	0.40	<.001		
Intercept	0.82	0.070	<.001	0.28	0.052	Intercept	28.36	1.21	<.001	83.64	19.02
Time	−0.036	0.004	<.001	0.0009	0.0026	Time	−0.77	0.088	<.001	0.22	0.10

Abbreviations: BDI‐II, Beck Depression Inventory second edition; Depression previous session, BDI‐II total score minus suicide item—1 week; Suicidality previous session, BDI‐II suicide item—1 week; Time, time was centered at the end of treatment (session 20); Var., variance.

## 4 DISCUSSION

The present study aimed to investigate the clinical effects of CT and IPT for MDD on SI. We found that the active conditions showed a greater decrease in SI compared with WLC as measured by the BDI‐II suicide item in a clinically depressed population. There were no differential effects in SI reductions between the two treatments, and the treatment effects of CT and IPT on decreasing SI remained significant after adjusting for change in overall depressive symptoms per session. Moreover, we found that SI measured at the previous CT or IPT session significantly predicted depressive symptoms at the current session. This temporal association was unidirectional, as we did not find depressive symptoms predicting SI in the course of therapy.

The finding that both CT and IPT reduced SI directly adds to the emerging literature showing that depression treatments not only reduce depression but also have an independent effect on SI. Earlier findings of Weitz et al. (2014) showed that the effect of depression treatment on change in suicidality disappeared once change in depression posttreatment was controlled for and suggested that depression might mediate suicidality. Our study controlled for other depressive symptoms at each therapy session and, contrary to Weitz et al. (2014), we found that the effects on SI remained significant. While initial baseline BDI‐II suicide item scores were similar in both studies, Weitz et al. (2014) focused on only two time points (pre‐ and posttreatment) rather than on the dynamic course of SI session‐by‐session during treatment. This means that even when controlling for depression at each session, CT and IPT have a direct effect on SI. Thus, instead of being a mere by‐product of depression that changes along with overall depression severity, SI represents an independent phenomenon impacting depression symptoms during treatment.

The efficacy of CT on this independent phenomenon (suicidality) was not completely unexpected (Miller et al., 2017; Rudd et al., 2015; Stanley et al., 2018) given the existence of effective brief variants of CT or CBT for suicide prevention and procedures (Bryan, 2019; Wenzel et al., 2009). Potentially, CT identifies and changes maladaptive cognitions, beliefs such as hopelessness, entrapment, defeat and self‐hatred, and self‐statements that contribute to suicidal behaviors (Bryan, 2019). Evidence for IPT on SI, however, has been scarce (Tang et al., 2009; van Orden et al., 2016), and the findings of Weitz et al. (2014) could not be attributed to the treatment itself since the decrease in SI was not specific for the psychotherapy conditions, but also occurred in the control groups (including placebo).

The current study was the first to imply that IPT for depression might be an effective treatment for SI as well. In line with the Interpersonal Theory of Suicide (Joiner, 2005), a possible underlying working mechanism of IPT for depression and its effects on suicidality could be that it targets two important interpersonal constructs of suicidality: thwarted belongingness (TB) and perceived burdensomeness (PB). TB occurs when the fundamental need for connectedness (i.e., “the need to belong”) is unmet. PB occurs when the need for social competence (i.e., “others would be better off without me”) is unmet (van Orden et al., 2010). The presence of a two‐way interaction between both constructs in combination with a state of hopelessness is assumed to lead to suicidal behaviors (van Orden et al., 2016). IPT targets these dynamic cognitive‐affective states by improving interpersonal social functioning and reduce interpersonal stressors (Hallensleben et al., 2019). However, more research on the underlying working mechanisms of depression treatments and their effects on SI is imperative.

Another critical finding in this study was that SI assessed at the previous session predicted depressive symptoms at the current session. This was not the case the other way around, as depressive symptoms of the previous session did not predict current SI. In a cross‐sectional data network analysis, depressive symptoms were one of the most important predictors of current SI (Beurs et al., 2019), but our findings examined the temporal relation over the course of therapy. This temporal relation suggests that SI may be a marker/risk factor for depression, supporting the idea that treatment may be most effective when directly targeting suicidal behavior or thoughts (Mewton & Andrews, 2016). This is in contrast with the frequently shared belief of clinicians that treating the other depressive symptoms will result in a positive effect on the suicidal symptoms (Sudak & Rajalakshmi, 2018). A previous multiple time‐point network analysis using this study sample (Bringmann et al., 2015), already indicated that suicidality (as measured by the BDI‐II suicide item) has the strongest negative impact on other depressive symptoms, but the other symptoms do not necessarily have a strong impact on SI. We built on this finding by showing that the SI marker seems to be an important *predictor* of future depressive symptoms.

### 4.1 Methodological considerations

By using multiple assessments of depression and SI and mixed‐models analyses, we were able to look at the dynamics and fluctuation of SI during a depression treatment. A strength of this study was the initial comparison with an untreated WLC condition. While it could be that the WLC condition showed a nocebo effect and performed worse than a true ‘natural course’ as they were aware they were waiting for a treatment, we did not see a deterioration in any symptoms. There are also some limitations. First, our sample is limited to a mild SI representation with no reported suicide attempts due to the design of our study. Given that our exclusion criteria were ‘high acute suicidal risk’ and comorbid substance abuse as well as the presence of an initial WLC, the more at‐risk suicidal individuals were probably not applied for participation and were not included. In this study, there were no reported adverse events of suicide attempts or completions. Second, overall baseline scores on the suicide item were low. This might have created a floor effect, impeding the detection statistically significant results. Furthermore, participants in the active conditions completed the BDI‐II every session, while the WLC did not complete additional questionnaires during the 2‐month waiting period. This may have created a training effect, which we were unable to analyze in our current data sample. Note, however, that suicide studies that evaluated the effects of repeatedly asking about suicide did not find a change in overall suicidality (Blades et al., 2018; Law et al., 2015; Polihronis et al., 2020). Third, there was no placebo group. While Weitz et al. (2014) showed significant decreases in SI, they did so in all treatment groups including the placebo group. Furthermore, while each participant on average had 17 repeated measures, we think that larger sample sizes of individuals with a wider range of suicidality as measured by a multiple‐item suicide instrument are warranted, as changes in SI were harder to detect with low baseline scores on a single item.

### 4.2 Clinical implications

In line with SI treatment guidelines (Bernert et al., 2014; Jacobs et al., 2010), our study results confirm the effectiveness of two widely implemented psychotherapies for depression (CT and IPT) on SI of a depressed patient. More importantly, as SI seems to be an independent phenomenon, and SI of the previous session appears to predict depressive symptoms at the current session, it may be most beneficial to treat SI first and foremost. Therefore, our study adds to the existing literature (Meerwijk et al., 2016; Sudak & Rajalakshmi, 2018; von Brachel et al., 2019) suggesting that the commonly held assumption that by the depression a decrease in suicidality in depressed patients will automatically follow from that is not supported.

### 4.3 Future directions

Given that, this is the first study that shows the effect of psychotherapy for depression on the dynamic of SI in the course of therapy, future research is needed to replicate these explorative findings. Moreover, since our sample was limited to a mild SI representation, future research may investigate the effects of CT and IPT in a population with a wider range of SI as measured by multiple‐item suicide instruments. Moreover, this study only focused on the treatment phase of both treatments. Future research could examine whether treating SI first could potentially also lead to a decrease in other *depressive* symptoms given the strong impact of SI on other depressive symptoms, and may examine the potential long‐term effects of these treatments on SI.

## CONFLICT OF INTERESTS

The authors declare that there are no conflict of interests.

## AUTHOR CONTRIBUTIONS

Marcus J. H. Huibers and Frenk F. P. M. L. Peeters designed the study. Lotte H. J. M. Lemmens, Marcus J. H. Huibers, and Frenk F. P. M. L. Peeters were responsible for the collection and management of study data. Suzanne C. van Bronswijk, Jaël S. van Bentum, and Marjan Drukker drafted the analysis plan and did the statistical analysis. Jaël S. van Bentum wrote the first draft of the manuscript. All authors were involved in data interpretation, critically revising the manuscript and approved the final version.

## Supporting information

Supplementary information.

## Data Availability

The data that support the findings of this study are available from the corresponding author upon reasonable request.
