# Supplementary material for: Cognitive therapy and interpersonal psychotherapy reduce suicidal ideation independent from their effect on depression
Source: Depress Anxiety. 2021 Mar 23;38(9):940–9. doi: 10.1002/da.23151 (PMC8451935; doi:10.1002/da.23151)
Supplement: Supplementary file 1 — Supplementary information. [file DA-38-940-s001.docx]

**SUPPLEMENTARY DATA**

**DATA SUPPLEMENT I: Detailed procedure of statistical analyses**

**Statistical analyses**

We started by exploring pre-treatment patient sociodemographic characteristics and clinical variables of the three condition groups: Cognitive Therapy (CT), Interpersonal Psychotherapy (IPT), and waiting-list control condition (WLC). We used descriptive statistics stratified by suicidal ideation (0 vs. > 1 on the BDI-II suicide item) and condition (n = 182).

Subsequently, we used an independent samples t-test (two-sided) to examine whether the active treatment groups (CT and IPT combined) outperformed the WLC condition by comparing change in BDI-II suicide item scores of patients in the CT and IPT groups after 8 weeks of therapy on the one hand to the WLC after 8 weeks of no treatment on the other hand.

Before we decided whether or not we could exclude all participants that scored 0 on the BDI-II suicide item at baseline, we wanted to check if suicidality scores of the patient population would fluctuate over the course of therapy. To test this, we examined the scores on the BDI-II suicide item for each session and checked if there were big fluctuations in number of participants with scores of 1 or higher on the BDI-II suicide item. Results showed that of those 60 participants, the scores of only 6-7 participants fluctuated. The rest of the sample’s scores remained at 0, thus we choose to only include patients with an increased level of suicidal ideation in any further analysis. A previous paper with similar analyses (Weitz et al., 2014) excluded non-suicidal participants from their sample as well.

From then onwards, we also limited the sample to only the active treatment groups (CT and IPT) since we were interested in the effects of treatment for depression on suicidality (n = 91). The participants in the WLC condition did not receive treatment as of yet.

To analyze the associations between depression treatments (CT and IPT) and suicidality change, repeated BDI-II suicide item scores at baseline and at the start of each therapy session were used as the dependent variable using mixed-effects (multilevel) models (Diggle et al., 2002). All analyses were intention-to-treat, meaning that all patients that enrolled in the study were included in the analyses, irrespective of completing therapy or assessments (Hollis & Campbell, 1999).

At first, the linear mixed-effects models had 3 levels: within-subject (level 1), between-subject (level 2), and between-therapist (level 3). However, the 3-level models failed to converge, similar to analyses performed by van Bronswijk and colleagues (van Bronswijk et al., 2018), most likely because of the relatively small number of participants per therapist. Thus, 2-level models were used instead and the therapist effect was analyzed in all models in the sensitivity analyses. Different transformations of time (linear, quadratic, loglinear) were assessed for each model with fit indices and visual inspection, and the linear model was determined as the best model fit.

The linear mixed-effect model included the following fixed effects: time (coded in terms of the session number), treatment (centered at CT = -0·5 or IPT = +0·5) and the other depressive symptoms (total BDI-II score excluding suicide item at each session) as a time varying co-variate. The difference between CT and IPT was represented by the time x condition interaction in the model. For the time variable, endpoint (session 20) was coded at zero. Moreover, intercepts and slopes (for the time variable) were allowed to vary randomly over subjects (and were allowed to be correlated). Residuals were allowed to be correlated using a time autoregressive structure and the exact time in weeks to each session was used to correct for the irregularly spaced time lags between the sessions (Jones, 1993). In order to examine whether CT and IPT differed in the change of suicidality levels over time as measured with the BDI-II suicide item, mixed (multilevel) regression analysis using restricted maximum likelihood estimation was used (see Figure 3).

Next, we performed two mixed-effects time-lagged models to examine whether there was a temporal relation between the change in depressive symptoms (BDI-II total score minus suicide item) and the change in suicidality (BDI-II suicide item). In the first model, we examined whether depressive symptoms of the previous session (depression – 1 week) predicted current suicidality. This model included the following fixed effects: time (number of sessions), and depression score at the previous session (as the lagged predictor variable). In the second model, we examined whether suicidality measured at the previous session (suicidality – 1 week) predicted current depressive symptoms. For that model, we included the fixed effects: time (number of sessions), and suicide item score of previous session (as the lagged predictor variable). All mixed analyses were performed using STATA (version 16.0). Other analyses were carried out in SPSS (version 25).

**References supplementary material**

Diggle, P., Heagerty, P., Liang, K., & Zeger, S. (2002). *Analysis of Longitudinal Data (Oxford Statistical Science Series)* (2nd ed.). Oxford University Press.

Hollis, S., & Campbell, F. (1999). What is meant by intention to treat analysis? Survey of published randomised controlled trials. *British Medical Journal*, 670–674.

Jones, R. (1993). *Longitudinal data with serial correlation: A State-Space Approach*. Chapman & Hall.

van Bronswijk, S. C., Lemmens, L. H. J. M., Viechtbauer, W., Huibers, M. J. H., Arntz, A., & Peeters, F. P. M. L. (2018). The impact of personality disorder pathology on the effectiveness of Cognitive Therapy and Interpersonal Psychotherapy for Major Depressive Disorder. *Journal of Affective Disorders*, *225*, 530–538. https://doi.org/10.1016/j.jad.2017.08.043

Weitz, E., Hollon, S. D., Kerkhof, A., & Cuijpers, P. (2014). Do depression treatments reduce suicidal ideation? the effects of CBT, IPT, pharmacotherapy, and placebo on suicidality. *Journal of Affective Disorders*, *167*, 98–103. https://doi.org/10.1016/j.jad.2014.05.036
